# Patient with rheumatoid arthritis with deep vein thrombosis presenting as a calf strain: a case report

**DOI:** 10.1186/s13256-020-02441-6

**Published:** 2020-07-24

**Authors:** Trevor Lewis

**Affiliations:** Extended Scope Physiotherapy Practitioner MSc MCSP, Physiotherapy Department, Aintree University Hospital, Liverpool, UK

**Keywords:** Rheumatoid arthritis, Calf strain, Deep vein thrombosis

## Abstract

**Background:**

Patients with rheumatoid arthritis experience various comorbidities, including cardiovascular disease. More data and awareness exist regarding the adverse effects of rheumatoid arthritis affecting the arterial side of the cardiovascular system, such as stroke or myocardial infarction, than regarding adverse venous complications, such as deep vein thrombosis and pulmonary embolism. Rheumatoid arthritis affects more women than men, and the risk of venous thromboembolism in rheumatoid arthritis tends to increase with age; therefore, the presentation in this case report of deep vein thrombosis in a nonsmoking, young, fit man with rheumatoid arthritis is rare. This patient was sent away from a minor injuries unit with a diagnosis of a calf strain. Further assessment at an accident and emergency department later in the day confirmed deep vein thrombosis via ultrasonography. This case report underlines the need for vigilance because deep vein thrombosis is a risk factor in rheumatoid arthritis, even in young, male, and physically fit individuals.

**Case presentation:**

A nonsmoking 39-year-old Caucasian man with a 2-year history of rheumatoid arthritis presented for assessment at a private physiotherapy clinic with a 4-week history of right-sided posterior calf pain that had developed following exercise at a gym. The patient therefore believed his symptoms were due to a calf strain. Findings at physiotherapy assessment suggested that the actual cause of the patient’s symptoms were as a result of deep vein thrombosis. The patient was directed to a local minor injuries unit with a referral letter from the author outlining this diagnosis. Following clinical assessment at the minor injuries unit, the patient was told that there was no likelihood of deep vein thrombosis, and his diagnosis was a calf strain. The patient harbored concerns at this point and decided to seek further medical opinion at a nearby accident and emergency department, where deep vein thrombosis was diagnosed using ultrasonography, and the patient was commenced on anticoagulants.

**Conclusions:**

Venous thromboembolism risk in rheumatoid arthritis is stated as being less recognized as an arterial complication. This is borne out by this patient’s clinical journey, wherein his youth, fitness, athletic appearance, and onset of symptoms during exercise were said to suggest a diagnosis of a calf strain at a minor injuries unit. Ultrasonography at a different accident and emergency unit later that day ultimately was used to diagnose deep vein thrombosis.

## Background

Rheumatoid arthritis (RA) is a common, chronic, and systemic inflammatory autoimmune disease; patients with RA have significant associated morbidity and shorter life expectancy than the healthy population [1]. Although RA primarily targets the synovial lining of the joints, it also has adverse extra-articular effects, including those affecting the pulmonary, cardiac, and vascular systems, which are said to contribute to shortened life expectancy [2]. In chronic inflammatory diseases such as RA, systemic inflammation and a hypercoagulable state are promoted with the upregulation of procoagulants, the downregulation of anticoagulants, and suppression of fibrinolysis [3], along with endothelial dysfunction [4].

There is said to be a paucity of data regarding the risks of RA-related deep vein thrombosis (DVT) or pulmonary embolism (PE) compared with the volume of research investigating the adverse effects of RA on the arterial system, such as myocardial infarction (MI) and stroke. There is also said to be less awareness of DVT and PE risk in RA among some practitioners [1, 2], even though this has been widely reported [5, 6].

PE is the third most common cardiovascular (CV) risk behind MI and stroke. There is a 15% fatality rate [1]; therefore, it is of vital importance to be aware of the increased venous thromboembolism (VTE) risk in RA, particularly when patients present with calf discomfort or any other symptoms that could implicate the CV system.

This report presents a case of a patient with RA who presented for assessment at a private physiotherapy clinic with symptoms that the patient himself thought were due to a calf strain but were suspected by the assessing physiotherapist of being as a result of DVT. The physiotherapist referred the patient directly to a local minor injuries unit with a supporting letter stating a clinical diagnosis of DVT and requesting further tests such as a D-dimer assay or an ultrasound scan to assess for this. The referral letter from the physiotherapist stated that the patient had a past medical history of RA, a known risk factor for DVT, and the awareness of this underpinned the presumptive diagnosis. At the minor injuries unit, the patient received clinical assessment (pulse, 66 beats/minute; blood pressure, 125/74 mmHg; temperature, 37.3 °C) and also calf squeezing and palpation. He was then told by the attending clinician that because he was young, fit, nonsmoking, healthy, and of athletic build, he was deemed to be unlikely to have suffered DVT, particularly because the onset of his calf pain was while exercising, so he was, therefore, sent away with a diagnosis of calf strain. The patient continued to harbor concerns that he may have a DVT and decided to seek a third opinion at an accident and emergency (A&E) department, where DVT was diagnosed using ultrasonography. The lack of awareness among some practitioners of the VTE risks in patients with RA, as stated in the literature, was evident in this patient’s experience at the minor injuries unit. The worst-case scenario was the patient accepting the diagnosis of a calf strain and potentially suffering a fatal PE.

## Case presentation

A 39-year-old Caucasian man who was an information technology operative (height, 182 cm; weight, 84 kg; body mass index, 25.4 kg/m^2^) attended a private physiotherapy clinic with a 4-week history of pain in the upper right calf. The patient is a nondrinker and has never smoked. The patient has no family history of RA or vascular disorders. The patient uses a standing desk when at work and regularly attends a gym, undertaking weight training plus CV exercise on various types of apparatus three times each week. The patient’s calf symptoms occurred during a gym session in which he was exercising on a step machine against high resistance, which resulted in his forcefully pushing up onto his toes; he therefore ascribed his symptoms to a calf strain. The patient’s only past medical history of note was a diagnosis of RA made 2 years previously. At onset, the patient’s RA had presented in a nonsymmetrical joint pattern affecting all metacarpophalangeal and metatarsophalangeal joints on the left. The patient’s rheumatologist prescribed the disease-modifying antirheumatic drug (DMARD) methotrexate at a dosage of 20 mg each week (eight 2.5-mg tablets once per week).

Upon clinical examination at the physiotherapy clinic, it was clear that the possibility of DVT of the right calf existed. This was based on the awareness that a diagnosis of RA carried an increased risk of CV complications. Moreover, the patient’s right calf was warm, and the area of tenderness was adjacent to the popliteal crease rather than the common area of tenderness of a calf strain that normally resides at the medial gastrocnemius muscle–tendon junction with the Achilles. The proximal site of the area of discomfort ruled out the distally situated soleus muscle; furthermore, resisted ankle plantar flexion produced no discomfort, which would normally be observed in the event of a posterior calf strain. This finding also ruled out the plantaris muscle as the source of the patient’s symptoms of injuries that can cause pain in the popliteal region.

The test result for Homans sign, a clinical test for DVT, was positive. At the end of physiotherapy assessment, the patient was told that his presentation was one of a possible DVT, and it was carefully explained that there were diagnostic limitations of clinical assessment in isolation for this pathology. Therefore, the patient was sent with a letter, clearly stating this clinical diagnosis, to a nearby minor injuries unit as a matter of urgency. The patient was clinically assessed by a member of the staff at the minor injuries unit who expressed doubt that calf thrombosis was the basis of his symptoms, stating that they would be “amazed” if the diagnosis was one of a DVT rather than a calf strain, basing this belief on the facts that the patient was young, male, healthy, of athletic build, and had first experienced his calf symptoms while using the stair-climber apparatus in the gym. The patient stated that he remained concerned that DVT rather than a calf strain was the diagnosis and that he wished to seek a further opinion, particularly because no investigations had been undertaken at this point. The patient was then directed to an A&E department 12 miles from the minor injuries unit. The patient directly attended this A&E department, where he was clinically assessed and underwent ultrasonography of the right calf. A DVT of the popliteal vein was diagnosed. The patient received an injection with heparin and was prescribed the anticoagulant apixaban at a dose of 10 mg twice daily for 7 days to be followed by a maintenance dose of 5 mg twice daily, and he was discharged from the A&E unit for his treatment to be managed in primary care. The patient was maintained on this dose of apixaban for 6 months, then he was reviewed by his general practitioner. At that time, the patient had experienced no adverse effects of this medication, such as headaches, visual impairment, or serious bleeding, and a dose of a 2.5-mg tablet of apixaban twice daily was decided on as a continued daily dose to reduce any future DVT risk.

## Discussion

This case is worthy of being reported because there is said to be a lack of awareness of the relationship between RA and increased risk of DVT [1, 2], and this assertion would appear to be supported by this patient’s experience at the minor injuries unit. To miss the diagnosis of VTE is potentially fatal; clinicians must keep in mind the possibility of a DVT with every patient who presents with posterior leg pain.

RA is a common disorder, but the cause of this disease is not known. Many possible etiologies have been identified. The annual incidence of RA has been reported to be approximately 40 per 100,000 population. The disease prevalence is about 1% in Caucasians, varying between 0.1% in rural Africans and 5% in certain groups of Native Americans. Women are affected two to three times more often than men [7, 8].

The connection between RA and VTE is said to be poorly understood, but it is thought that in patients with RA, systemic inflammation and production of cytokines such as tumor necrosis factor-α and interleukin 1 precipitate a state of hypercoagulability. These proinflammatory cytokines potentiate vascular endothelial dysfunction, inhibition of fibrinolysis, and downregulation of protein C [9].

The possibility also exists that patients with RA have increased VTE risk due to surgical procedures associated with the condition, acute hospitalization, reduced activity levels, and CV comorbidity.

The increased risk of VTE has been investigated by numerous researchers. One group of investigators [6] analyzed a national health database in Taiwan and found that when patients with RA (*n* = 29,238; 77% women; mean age, 52.4 years) were compared with age- and sex-matched control participants from the general population (*n* = 116,952), there was a 3.36-fold increase in the risk of developing DVT and a 2.07-fold increase in the risk of developing PE in patients with RA compared with patients without RA, and this risk was significantly increased in the first 4 years following RA diagnosis. This study also found that VTE development increased with age in both RA and non-RA cohorts. Similar findings were reported when collecting data from 1986 to 2010 [2] in an investigation of 9589 patients with RA (69% female; mean age, 58 years) compared with non-RA individuals (*n* = 95,776). These researchers observed an increased risk of PE and DVT in RA. These investigators also noted a sixfold increase in the risk of PE during the first year following any hospital admission in patients with autoimmune disorders; this is line with similar observations by other researchers [10, 11].

The patient in this case report had been diagnosed with RA 2 years previous to the onset of his calf symptoms; he had not been hospitalized since his diagnosis of RA. He presented with a subjective history of what he believed to be a calf strain, but this was atypical for a muscular injury due to the fact that there was no tenderness, bruising, or ecchymosis at any of the common sites of muscle strain in the calf. There was only a warm and slightly swollen region just distal to the popliteal crease. The Homans sign, which involves eliciting calf pain in the event of DVT using forced dorsiflexion of the ankle on the affected side, was traditionally accepted to be a reliable test, but its sensitivity and specificity as a clinical test have been queried more recently due to the fact that this test generates pain in the event of a calf strain in the absence of a DVT [12]. The result of the Homans sign test was positive in this patient; however, resisted plantar flexion of the ankle on the symptomatic side was not painful, which is atypical of a posterior calf strain [13].

Baker’s cysts can be misinterpreted as DVT [5]. Although patients with RA are at increased risk of developing Baker’s cysts, there was no clinical sign of such a structure in this patient. The patient had received a diagnosis of RA 2 years previously, and the author’s awareness that this disease presents an increased risk of VTE contributed to the clinical suspicion that DVT could be present.

Since the patient’s diagnosis of RA, he had been prescribed and taking DMARDs in the form of methotrexate, which, along with glucocorticoids and nonsteroidal anti-inflammatory drugs, are commonly used in the treatment of RA but are also recognized as carrying a risk of causing DVT [2].

## Conclusions

There is a growing body of evidence that RA and other autoimmune diseases carry an elevated risk of VTE in the form of DVT and PE. This risk is particularly elevated in the first 4 years after diagnosis of RA, and DMARDs, which are commonly used to treat RA, are also said to present an elevated risk of DVT. The VTE risk tends to increase with age and any hospital admissions. RA also affects more females than males; therefore, a nonsmoking, otherwise fit and lean, 39-year-old man with RA with no hospital admissions since diagnosis presenting with calf pain following a gym session posed a clinical dilemma wherein a muscular strain may have been accepted as a diagnosis. This is evidenced by the fact that a muscular injury to the calf was the conclusion arrived at when the patient attended a minor injuries unit despite having been sent there directly following physiotherapy assessment with a letter of referral outlining a strong suspicion of DVT. Had the patient taken the advice given at this point in his clinical pathway, and had he not persisted in attending the A&E unit, grave illness or fatality could have resulted. The risk of VTE should be carefully considered in patients with RA who present with any potential CV signs, including calf pain or discomfort. This case report outlines that there is a need for vigilance for the possibility of VTE in patients with RA.
